# Identification of excitatory premotor interneurons which regulate local muscle contraction during *Drosophila* larval locomotion

**DOI:** 10.1038/srep30806

**Published:** 2016-07-29

**Authors:** Eri Hasegawa, James W. Truman, Akinao Nose

**Affiliations:** 1 Department of Complexity Science and Engineering, Graduate School of Frontier Sciences, The University of Tokyo, Kashiwanoha, Kashiwa, Chiba, Japan; 2 Janelia Research Campus, Howard Hughes Medical Institute, Ashburn, Virginia, The United States of America; 3 Department of Physics, Graduate School of Science, The University of Tokyo, Hongo, Bunkyo-ku, Tokyo, Japan

## Abstract

We use *Drosophila* larval locomotion as a model to elucidate the working principles of motor circuits. Larval locomotion is generated by rhythmic and sequential contractions of body-wall muscles from the posterior to anterior segments, which in turn are regulated by motor neurons present in the corresponding neuromeres. Motor neurons are known to receive both excitatory and inhibitory inputs, combined action of which likely regulates patterned motor activity during locomotion. Although recent studies identified candidate inhibitory premotor interneurons, the identity of premotor interneurons that provide excitatory drive to motor neurons during locomotion remains unknown. In this study, we searched for and identified two putative excitatory premotor interneurons in this system, termed CLI1 and CLI2 (cholinergic lateral interneuron 1 and 2). These neurons were segmentally arrayed and activated sequentially from the posterior to anterior segments during peristalsis. Consistent with their being excitatory premotor interneurons, the CLIs formed GRASP- and ChAT-positive putative synapses with motoneurons and were active just prior to motoneuronal firing in each segment. Moreover, local activation of CLI1s induced contraction of muscles in the corresponding body segments. Taken together, our results suggest that the CLIs directly activate motoneurons sequentially along the segments during larval locomotion.

Animals perform various types of rhythmic movements such as respiration, chewing and locomotion for their survival. These rhythmic movements are thought to be regulated by neuronal circuits termed central pattern generators (CPGs)^1^,^2^,^3^,^4^. CPGs consist of interneurons and motoneurons whose rhythmic activities induce coordinated patterns of muscle contraction. Although CPGs are regulated by descending and sensory inputs, rhythms very similar to those seen in the intact animal can be generated without these inputs^1^,^2^,^3^. Because CPGs of invertebrates and vertebrates share many characteristics, CPGs in one animal could be a model for other animals^5^,^6^. Moreover, because CPGs show many characteristics common to other neuronal systems, CPGs could be a general model linking neuronal circuits to behaviour^5^. Despite the efforts to elucidate the function of CPGs, their identities and functional mechanisms are not completely understood, in particular in animals with a large central nervous system (CNS). This is partly because manipulating the function of specific neurons in the neural circuits is often difficult, especially in animals with vast numbers of neurons such as mammals.

The *Drosophila* larva is emerging as an excellent model system for studies of CPGs because one can use sophisticated genetic methods, such as the Gal4-UAS system^7^, to manipulate and visualize the activity of specific component neurons^8^ in a moderately sized CNS consisting of ~10,000 neurons^9^. Larval forward locomotion is executed by the sequential contraction of muscles from the posterior to the anterior segments^10^,^11^,^12^,^13^,^14^. Motoneurons in the ventral nerve cord (VNC) actualize the sequential muscle contraction by being activated from the posterior to the anterior segments during forward locomotion^15^,^16^,^17^. CPGs responsible for the locomotion seem to be present in the VNC, since neuronal circuits in the thoracic and abdominal segments have been shown to be sufficient for generating the behavior^18^,^19^. Calcium imaging of the entire CNS has visualized neurons that are active during larval locomotion including those in the brain, sub-oesophageal zone (SEZ), and the VNC^16^. However, the identities of these neurons are only beginning to be characterized^16^,^20^,^21^,^22^,^23^.

Previous studies showed that motor neurons in the VNC receive both excitatory and inhibitory inputs^24^,^25^. It is therefore likely that specific patterns of motoneuron activation are regulated by the balance and the timing of excitatory and inhibitory inputs as shown in other systems^2^,^3^,^4^. Recently, two types of inhibitory premotor interneurons that regulate larval locomotion have been identified. PMSIs (*period*-positive median segmental interneurons) are glutamatergic inhibitory premotor interneurons that regulate the speed of larval locomotion^20^. Another glutamatergic interneuron, GVLIs (glutamatergic ventro-lateral interneurons) seem to function as premotor inhibitory neurons to terminate motor bursting^21^. In contrast, premotor interneurons that provide excitatory inputs to motor neurons during locomotion remain to be identified, although they are known to be cholinergic^24^,^25^. A recent study identified two cholinergic descending interneurons that form putative synaptic contacts with segmental motoneurons^26^. However, whether they are active and play roles during locomotion remains unknown.

In the present study, we searched for and identified putative excitatory premotor interneurons that activate motoneurons during locomotion. These neurons, termed CLI1 and CLI2 (cholinergic lateral interneuron), are segmental interneurons that show wave-like activity during locomotion concurrent with the activity propagation of motoneurons. Consistent with CLIs being excitatory premotor neurons, these neurons form GRASP- and ChAT-positive synaptic contacts with motor neurons and are activated just before the activation of motoneurons in each segment. In addition, forced activation of these neurons locally induces the contraction of muscles. Our results suggest that wave-like activity of CLIs activates motoneurons sequentially along the segments during forward locomotion.

## Results

### Identification of interneurons showing wave-like activity

**Figure 1:**
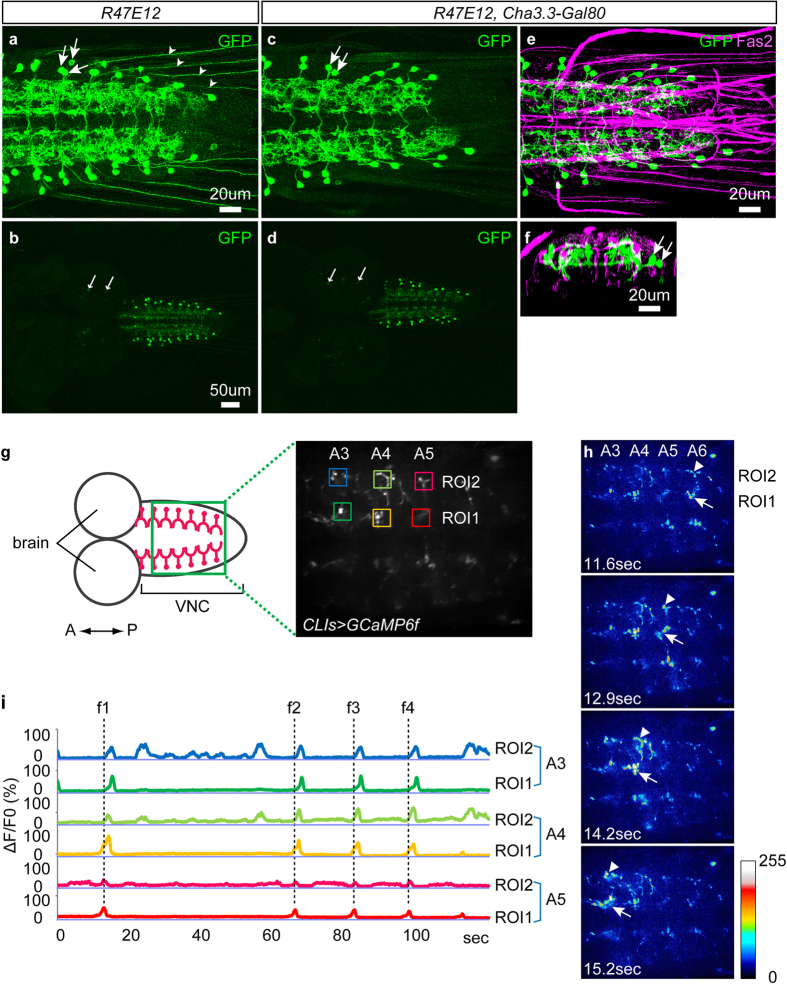
Identification of CLIs. (**a**–**f**) Expression of *R47E12-Gal4* (**a**,**b**) and *CLI1/2-Gal4* (*R47E12-Gal4, Cha-Gal80*) (**c**–**f**) visualized with the *UAS-mCD8GFP* reporter in third instar larvae. Stacked images of the VNC (**a**,**c**,**e**) and the entire CNS (**b**,**d**) are shown (dorsal view). A cross-sectional view of (**e**) is shown in (**f**). Fas2 expression (magenta) is also shown as a positional reference in (**e**,**f**). (**a**,**b**) *R47E12* drives expression in ~3 somas per hemisegment (arrows). It also drives expression in sensory neurons (small arrowheads) and in a few cells in the brain (small arrows). (**c**–**f**) *CLI1/2-Gal4* drives expression only in two interneurons per hemisegment, which we designated as CLI1 and CLI2, in the ventral nerve cord (arrows). It also drives expression weakly in a few cells in the brain (small arrows in **d**). (**g**–**h**) Calcium imaging of *CLI1/2-Gal4* expressing neurons. (**g**) Schematic of the larval CNS and a maximum projection of imaged movies showing the regions of interests (ROIs) used for data analysis (coloured rectangles). A3–A5: abdominal segment 3 to 5. (**h**) Still images of activity propagation in the *Gal4* expressing neurons. Fluorescence change in the *Gal4* expressing neurons (shown with arrows and triangles) propagated from the posterior to the anterior segments. (**i**) Traces of fluorescence change in CLI1s and CLI2s are shown. The color of the traces corresponds to that of ROIs in (**g**). f1 to 4 indicate forward waves. Anterior is to the left in this and the following figures unless otherwise noted.

Neurons involved in rhythmic movements are likely to be activated in a rhythmic manner. We therefore searched for interneurons that show rhythmic activity, among the neurons targeted by the Janelia Research Campus Gal4 lines^27^. We first selected Gal4 lines that expressed Gal4 in small subsets of neurons in the VNC and then performed calcium imaging of Gal4-expressing neurons, using GCaMP6f 28 as a reporter. We report here on two cholinergic interneurons, cholinergic lateral interneurons 1 and 2 (CLI1 and CLI2), that we found to show wave-like activities during fictive locomotion in the isolated CNS^15^,^20^. These neurons were originally found among the neurons that are targeted by *R47E12-Gal4* (Fig. 1a,b). Since *R47E12-Gal4* expresses Gal4 in several types of interneurons, we used several Gal80 lines to narrow down Gal4-driven expression only in neuron(s) that show the rhythmic activity. We found that *Cha3.3-Gal80*^29^ confines *R47E12-Gal4*-driven expression in two pairs of neighboring neurons in each abdominal segment A1–A7 (Fig. 1c–f). As described below, these neurons were both cholinergic, shared similar morphological characteristics, and were simultaneously active during fictive locomotion. We therefore named these neurons as CLI1 and CLI2. Below we used the *Gal80*/*Gal4* combination (*Cha3.3-Gal80*, *R47E12-Gal4*; hereafter called *CLI1/2-Gal4*) to target both CLI1s and CLI2s. As shown in Fig. 1g–i, the neurites of these neurons showed a wave-like activity that propagated sequentially from the posterior to anterior segments during fictive locomotion in the isolated CNS. We also observed the wave-like activity in dissected larvae and confirmed that the activity occurs concomitantly with the wave of muscle contraction (Supplementary Video S1). Since the wave-like activities of these neurons were observed in the isolated CNS without sensory input, these neurons are likely a part of the CPG circuits.

### CLI1s and CLI2s are activated just prior to motoneurons in the same segment

**Figure 2:**
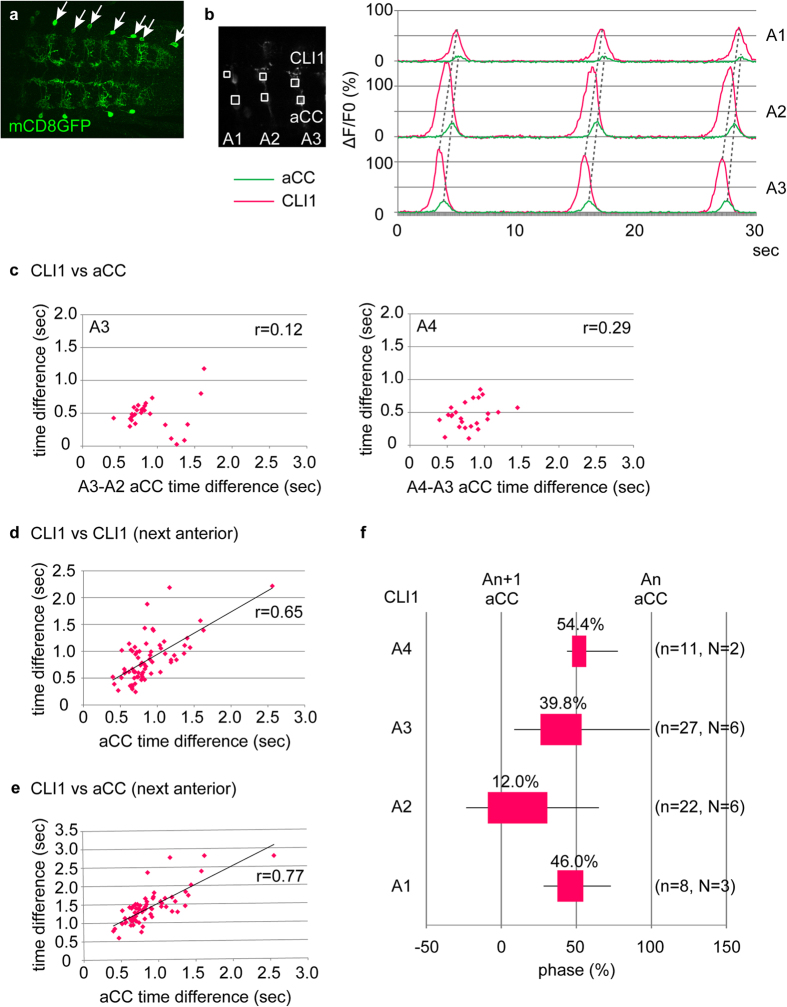
CLI1s are activated just before motoneurons during forward motor waves. (**a**) *CLI1-Gal4* (*tsh-Gal80; R47E12-Gal4, Cha-Gal80*) drives expression specifically in a pair of CLI1s in each abdominal segment (as visualized with *UAS-mCD8GFP*). (**b**) Calcium imaging of CLI1s and aCC. Maximum intensity projection of imaged movies showing ROIs (left), and the traces of fluorescence change in each ROI (right) are shown. (**c**) CLI1s are activated just prior to aCCs in the same segment. Time difference between the activities of CLI1 and aCC in the same segment is plotted against the intersegmental time delay (time difference between the activities of aCCs and those in the next anterior segment). A3, 27 waves from 6 samples; A4, 27 waves from 6 samples; r: Pearson’s correlation coefficient. (**d**,**e**) Time difference between CLI1s in neighboring segments (**d**, 71 waves from 15 samples) or that between CLI1s and aCCs in the next anterior segment (**e**, 68 waves from 15 samples) is proportional to the intersegmental time delay. (**f**) Phase representation of the activity timing of CLI1. Activity timing of CLI1s in each segment [A_n_; n = 1–4] normalized with that of aCCs in the same [A_n_] and next posterior [A_n + 1_] segment set as 100 and 0%, respectively. Activity timings are represented in box-and-whisker plots. Average phases are indicated above the boxes. Wave numbers (n) and sample numbers (N) are indicated in the parenthesis. The experiments were done using wandering third instar larvae.

**Figure 3:**
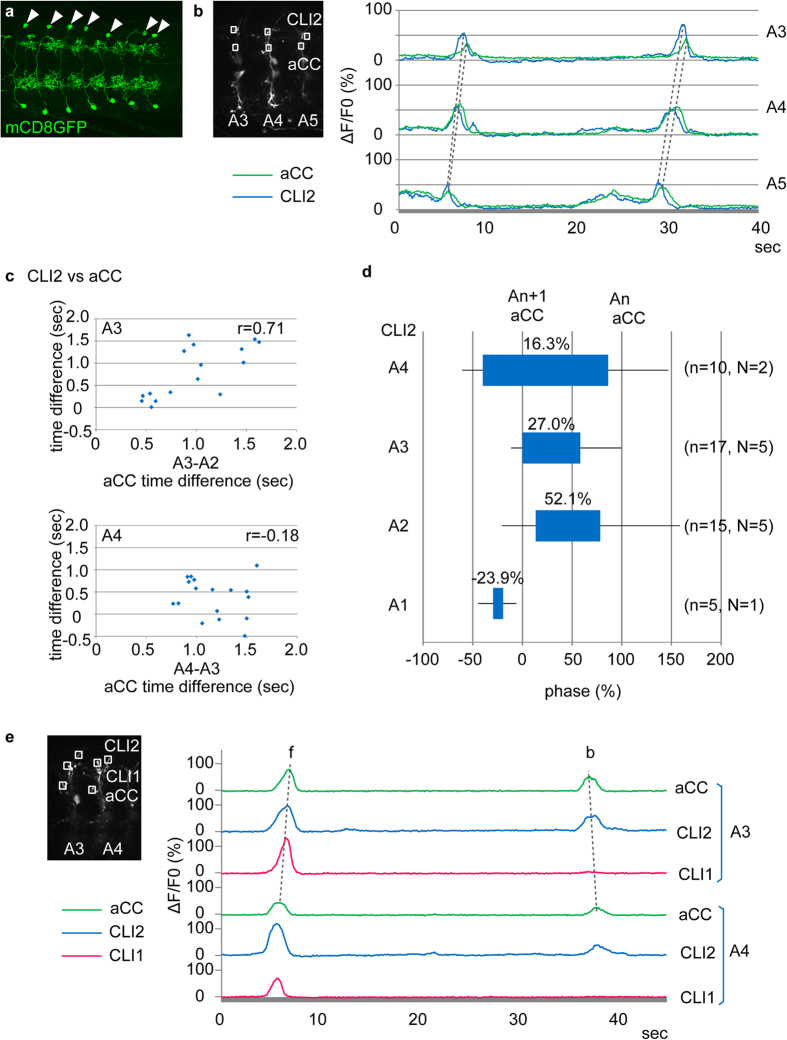
CLI2s are often activated just before motoneurons during both forward and backward motor waves. (**a**) *CLI2-Gal4* (CLI2 split-Gal4 line, *JRC*-*SS01256*) drives expression specifically in a pair of CLI2s in each abdominal segment (as visualized with *UAS-mCD8GFP*). (**b**) Calcium imaging of CLI2s and aCC. (**c**) CLI2s are activated just prior to aCCs in the same segment. Time difference between the activities of CLI1 and aCC in the same segment is plotted against the intersegmental time delay. A3, 16 waves from 5 samples; A4, 17 waves from 5 samples. (**d**) Phase representation of CLI2 activities. The activity timings are normalized as in Fig. 2f. (**e**) Calcium imaging of CLI1s, CLI2s and aCC during forward and backward waves. CLI1s are active only during forward waves, while CLI2s are active during both forward and backward waves. The experiments were done using wandering third instar larvae.

Since the neurites of CLI1s and CLI2s overlap, it was difficult to assign the wave-like activity described above to each individual neuron. We therefore searched for combinatorial Gal4/Gal80-expressions that are specific to each neuron. We found that *tsh-Gal80*^30^ suppresses *CLI1/2-Gal4-driven* expression in CLI2s but not in CLI1s. We therefore used the *tshGal80*, *CLI1/2-Gal4* combination to specifically target CLI1s (hereafter called *CLI1-Gal4*, Fig. 2a). We also searched for and identified split Gal4 lines specific to CLI2s (called *CLI2-Gal4-a to c*, Fig. 3a, Supplementary Fig. S1). Use of these CLI1- or CLI2-specific lines, as well as clonal analyses of CLIs (described below), allowed us to uniquely identify the neurites of CLI1s and CLI2s. We therefore used the original *CLI1/2-Gal4* together with a motor neuron driver, *RRa-Gal4-F*^31^, to examine the temporal correlation between the activities of each CLI and motoneurons (*CLI1/2-Gal4*, but not the CLI1- or CLI2-specific Gal4 lines, was used because the level of expression is higher in the former). Since *RRa-Gal4-F* drives expression only in two motor neurons, aCC and RP2, in each hemisegment, the dendrites of which can be readily distinguished from those of CLIs, we could simultaneously record the activity of the motor neurons and CLIs by expressing *GCaMP6f* in these neurons. As detailed below, we found that both CLI1s and CLI2s are active during fictive locomotion just prior to the motor neurons.

Figure 2 shows the activities of CLI1s in relation to those of aCCs. CLI1s were activated sequentially from the posterior to anterior segment at a similar timing as aCC motoneurons in the same segment in all forward waves examined (Fig. 2b, 100%, n = 96 waves). CLI1s were always activated just prior to aCCs in each segment at various speeds of fictive locomotion (Fig. 2c; Supplementary Fig. S2). When the time difference in the activity of CLI1s in two neighboring segments was plotted against the intersegmental time delay of motor activity, a linear relationship was observed, suggesting that these interneurons were part of intersegmental circuits that scale intersegmental delay with cycle period (Fig. 2d)^15^,^21^. Similarly, the time difference between CLI1s and aCCs in the next anterior segment was proportional to the intersegmental delay (Fig. 2e). In contrast, the time difference between CLI1s and aCCs in the same segment did not scale with the speed of fictive locomotion (Fig. 2c, Supplementary Fig. S2), suggesting that the activity timing of these neurons are tightly coupled regardless of the speed of locomotion. Having established the scaling of the activity of CLI1s with cycle period, we calculated phase relationships between the activity of CLI1 and aCC, and confirmed that CLI1s are activated at an earlier phase than aCCs in the same segment (Fig. 2f).

Like CLI1s, CLI2s were activated just prior to aCCs in the same segment in most of the forward waves at different speeds (68.8% [n = 16] in A2, 100% [n = 16] in A3, 76.5% in A4 [n = 17], 100% in A5 [n = 12]; Fig. 3b,c; Supplementary Fig. S2), although the time differences are more variable compared to that between CLI1s and aCCs. Thus, CLI2s’ activity is also correlated with motor activity in the same segment. The relative timing of the activity of CLI2s and aCCs was also confirmed in a phase representation (Fig. 3d). We also studied the activity of CLI1s and CLI2s during fictive backward locomotion and found that CLI2s but not CLI1s were recruited to backward locomotion (Fig. 3e; CLI1s and CLI2s were active in 3.2% (n = 31 waves) and 84.8% (n = 33 waves) of backward waves, respectively).

### CLI1s and CLI2s are commissural cholinergic interneurons present in each abdominal segment

**Figure 4:**
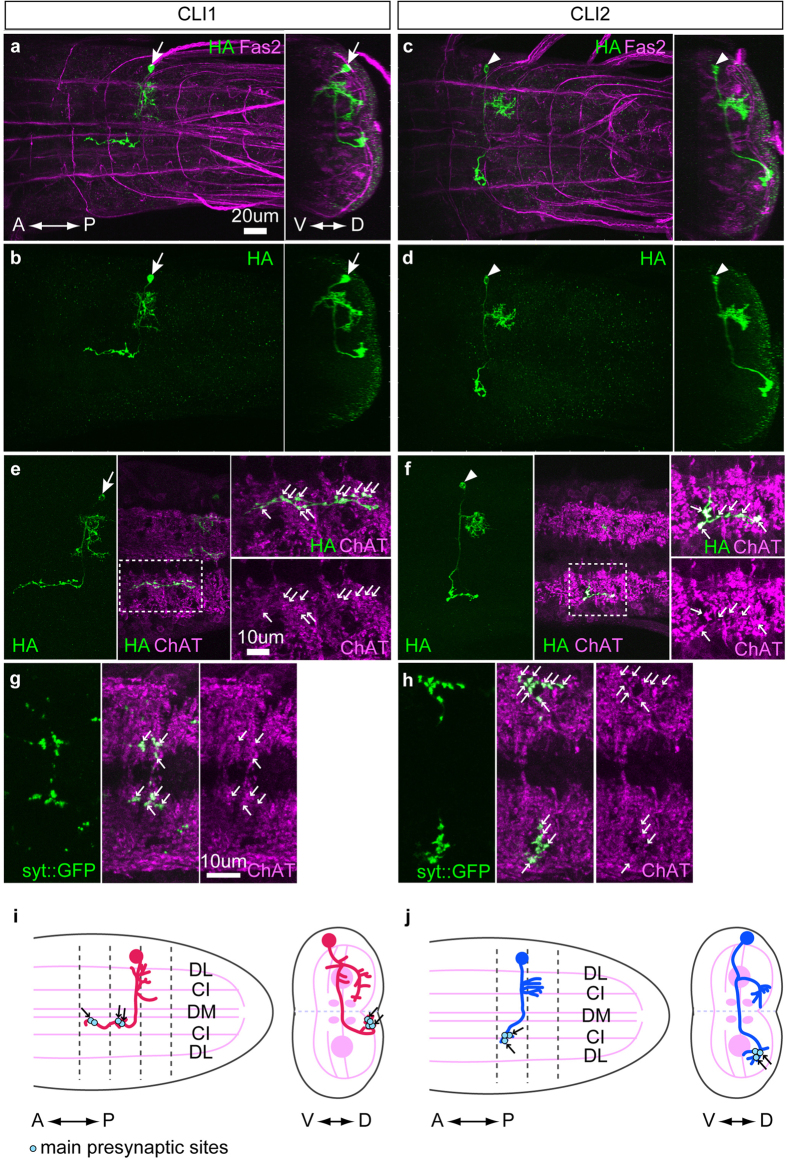
CLI1s and CLI2s are segmental cholinergic interneurons with contralateral projections. (**a**–**d**) Representative single neurons of CLI1s (**a**,**b**) and CLI2s (**c**,**d**) (visualized with HA, green) obtained by the MCFO method in wandering third instar larvae. Fas2 expression is also shown (magenta). (left) Dorsal view. (right) Cross-sectional view. (**e**,**f**) A single neuron of CLI1 (**e**) and CLI2 (**f**) co-stained for HA (green) and anti-ChAT antibodies (magenta). Stacked image (HA) and a single optical section of 0.98 um thickness (HA and ChAT) are shown. Regions shown in rectangles are enlarged in the right panels. Axon terminals of CLI1s and CLI2s contain puncta that co-localize with ChAT (small arrows). Positions of somas are indicated with arrows (CLI1s) and triangles (CLI2s). (**g**,**h**) Presynaptic sites of CLI1s and CLI2s were visualized by *CLI1* > *Syt1::GFP* (**g**) and *CLI2* > *Syt1::GFP* (**h**) (green), respectively, and co-stained with anti-ChAT antibodies (magenta). ChAT positive-puncta in CLI1s and CLI2s axon terminals are also Syt1-GFP positive (magenta). Stacked images of 7 (**g**) and 10 (**h**) confocal images are shown. (**i**,**j**) Schematic of CLI1s (**i**) and CLI2s (**j**). Fas2 coordinates are shown as landmarks. Segment borders are shown with dashed lines. ChAT positive putative presynaptic sites are indicated by small arrows. C: central, D: dorsal, I: intermediate, L: lateral, M: median, V: ventral, A: anterior, P: posterior.

We next performed single cell analysis to examine the morphology of individual CLI1s and CLI2s. We used multi-colour flp-out (MCFO) technique^32^ to visualize single neurons of CLI1s and CLI2s and characterized their morphology using the Fas2-coordinate as a positional reference^33^. CLI1s and CLI2s showed similar morphological characteristics: they both are located in a lateral region of the CNS and extended an axon to the contralateral side through the anterior commissure (Fig. 4a–d,i,j). However, their final axonal projection patterns were different. After crossing the midline, CLI1s innervated a medial region of the dorsal neuropile and further extended anteriorly toward the next segment. By contrast, CLI2s innervated a more lateral region in the dorsal neuropile and terminated within the segment in most of the cases (11/14 neurons examined) but occasionally projected to the next posterior segment (3/14). Arborization patterns of their putative dendrites were also distinct: dendrites of CLI1s were located mainly in the same segment with some in the next posterior segment, while dendrites of CLI2s were exclusively in the next posterior segment. Staining with anti-Choline acetyltransferase (ChAT) antibodies showed that the axon terminals of CLI1s and CLI2s contained ChAT-positive puncta (Fig. 4e,f). These ChAT-positive puncta were also positive for the presynaptic marker Synaptotagmin1::GFP^34^, indicating that they are presynaptic sites (Fig. 4g,h). Staining with anti-GABA or anti-vGluT did not show co-localisation with the soma or the terminals of CLIs (Supplementary Fig. S3).

### CLIs form putative cholinergic synapses with motoneurons

**Figure 5:**
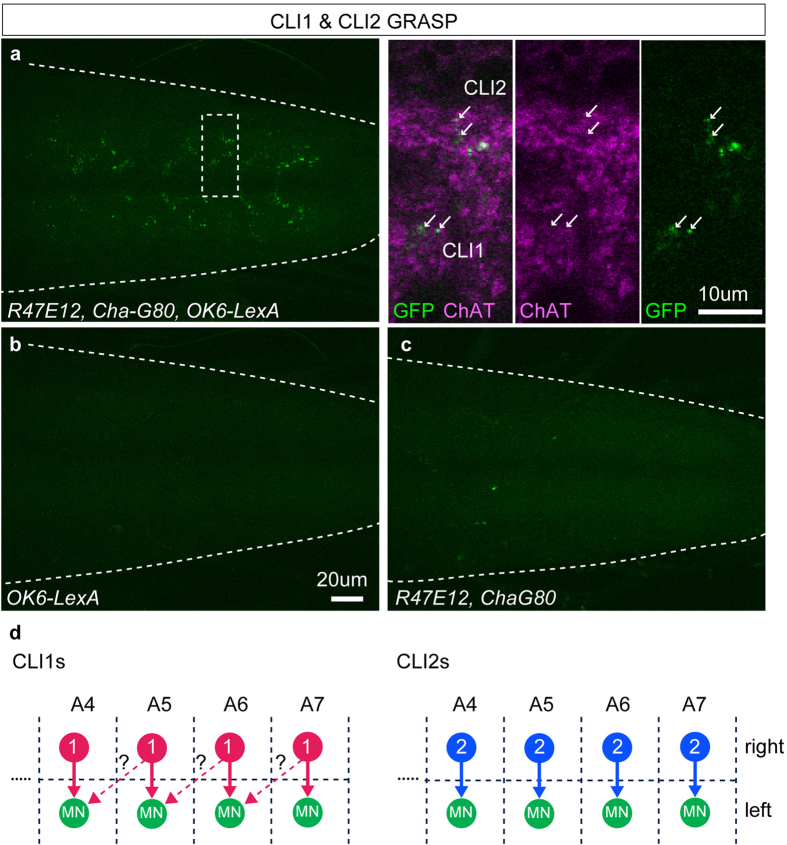
CLIs form GRASP-positive putative synapses with motoneurons. (**a**–**c**) Synaptic contacts with motoneurons were visualized using the GRASP method in wandering third instar larvae. (**a**) GRASP analysis with *CLI-Gal4* and *OK6-LexA*. A single optical section (0.98 um thickness) of a region shown in a rectangle is enlarged in the right panels. Punctate GFP signals, which co-localized with ChAT (small arrows), are observed in a region occupied by the axon terminals of CLIs. GRASP signals in the medial and lateral region likely correspond to the synapses of CLI1s and CLI2s, respectively. Genotype: *OK6-LexA, lexAop-CD4::spGFP11/OK6-LexA; UAS-CD4::spGFP1-10/R47E12-Gal4, Cha3.3-Gal80*. (**b**) Control of GRASP analysis without *R47E12-Gal4.* Genotype: *OK6-LexA, lexAop-CD4::spGFP11/OK6-LexA; UAS-CD4::spGFP1-10*. No signal is observed. (**c**) Control of GRASP analysis without *OK6-LexA.* Genotype: *lexAop-CD4::spGFP11; UAS-spGFP1-10/R47E12-Gal4, Cha3.3-Gal80*. No signal is observed. (**d**) Possible connection between CLIs and motoneurons. (left) CLI1s innervate and activate motoneurons in the same segment. CLI1s also project anteriorly, and may activate motoneurons in the next anterior segment. (right) CLI2s innervate and activate motoneurons in the same segment.

CLI1s and CLI2s innervate the dorsal neuropile where motor neuron dendrites are present. Previous studies had also shown that acetylcholine excites motor neurons^25^, raising a possibility that CLI1s and CLI2s are excitatory premotor neurons. To pursue this possibility, we first simultaneously visualized the axon terminals of CLIs and dendrites of motoneurons, and found that they are close to each other (Supplementary Fig. S4). We then used GRASP (GFP Reconstruction Across Synaptic Partners) method^35^,^36^ to test whether CLI1s and CLI2s form synaptic contacts with motoneurons. We used *CLI-Gal4* and *OK6-LexA* to express complementary split-GFPs in CLIs and motor neurons, and detected reconstituted GFP signals in ChAT-positive puncta in a region occupied by the presynaptic sites of CLI1s and CLI2s (Fig. 5a). No signal was observed in control larvae that express split-GFP only in CLI1s/CLI2s or in motoneurons, confirming the specificity of the GRASP signal (Fig. 5b,c). These results suggest that CLIs are premotor interneurons. To summarize the activity and anatomical data described above, CLI1s and CLI2s are cholinergic interneurons that are present in each abdominal segment and project their axons locally and contra-laterally to innervate motor neurons in the same segment. Since CLI1s send an intersegmental axon collateral, they may also innervate motor neurons in the next anterior segment (Fig. 5d).

### Forced activation of CLIs induces contraction of muscles

**Figure 6:**
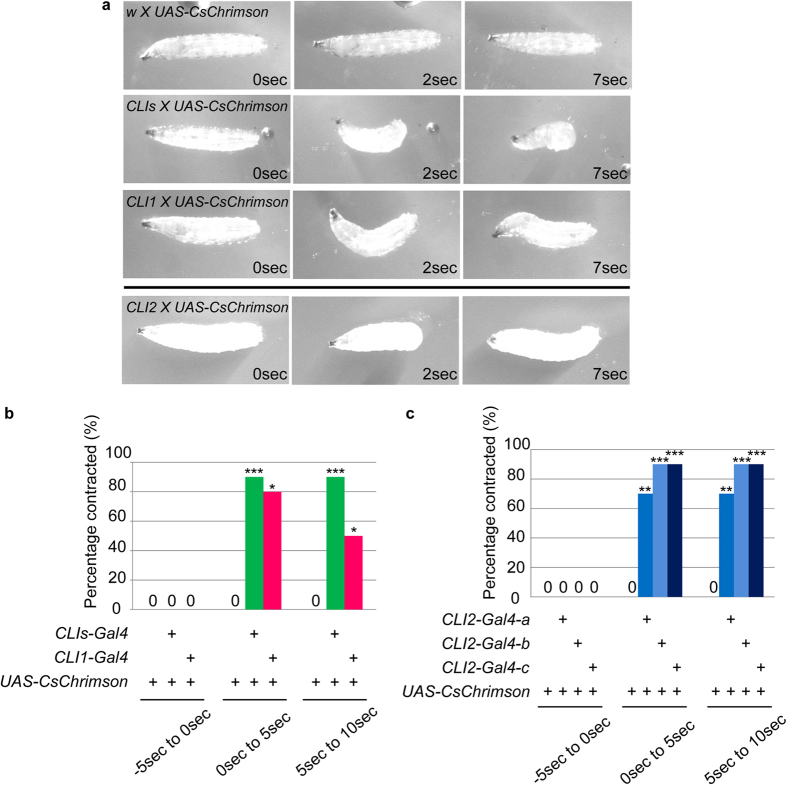
Forced activation of CLI1s and CLI2s in the larvae induces contraction of the body. (**a**) Still images of control and experimental larvae after light application. Third instar larvae expressing CsChrimson driven by *CLI1/2-Gal4*, *CLI1-Gal4*, *or CLI2-Gal4* contract their body when stimulated with light. (**b**,**c**) Percentage of animals showing body contraction in three 5-seconds time windows before and after light stimulation at time 0. *p < 0.05, ** < 0.01, *** < 0.005, Fisher’s exact test. N = 10 for each genotype. P-values were 0.0004, 0.0012, 0.0004, 0.049 from left to right in (**b**), 0.006, 0.0004, 0.0004, 0.006, 0.0004, 0.0004 from left to right in (**c**). Error bars indicate the SEM. Genotypes; *CLI1/2-Gal4*: *R47E12-Gal4, Cha-Gal80*, *CLI1-Gal4*: *tsh-Gal80; R47E12-Gal4, Cha-Gal80*, *CLI2-Gal4-a*; *JRC-SS01256*, *CLI2-Gal4-b*: *JRC-SS01809*, *CLI2-Gal4-c*: *JRC-SS01342*.

If CLI1s and CLI2s provide excitatory inputs to motoneurons, forced activation of these neurons may result in contraction of muscles. We tested this possibility by optogenetically activating CLI1s and/or CLI2s during locomotion. We used *CLI1/2-Gal4, CLI1-Gal4* and *CLI2-Gal4*, to express CsChrimson, a red-shifted version of channelrhodopsin^37^, in CLI1s and CLI2s, CLI1s, and CLI2s, respectively, and activated these neurons in the larvae undergoing locomotion. Application of red light to control larvae had no effect on the behavior of the larvae and they continued forward locomotion. However, when the light was applied to larvae expressing CsChrimson in CLI1s and/or CLI2s, the larvae contracted their abdomen and halted forward locomotion (Fig. 6a–c and Supplementary Videos S2,S3,S4,S5). Activation of CLI2s but not CLI1s also induced lifting of the tail (Supplementary Videos S4 and S5; 40% [n = 10] with *CLI2-Gal4-a*; 70% [n = 10] with *CLI2-Gal4-b*; 80% [n = 10] with *CLI2-Gal4-c*). Degree of contraction was stronger during the first 5 seconds of light application. Afterwards, the contraction gradually weakened. In the case of activating only CLI1s, some larvae were released from the contraction after 5 seconds of light application and resumed normal locomotion (Fig. 6b). Of note, activating CLI2s using three independent intersectional Gal4 lines resulted in the contraction of muscles (Fig. 6c). Since the only cells commonly targeted by the three Gal4 lines are CLI2s (Supplementary Fig. S1), the result indicates that activation of CLI2s was responsible for the muscle contraction phenotype. In the case of CLI1 activation, the involvement of other neurons targeted by *CLI1-Gal4* (a few cells in the brain) could not be excluded.

**Figure 7:**
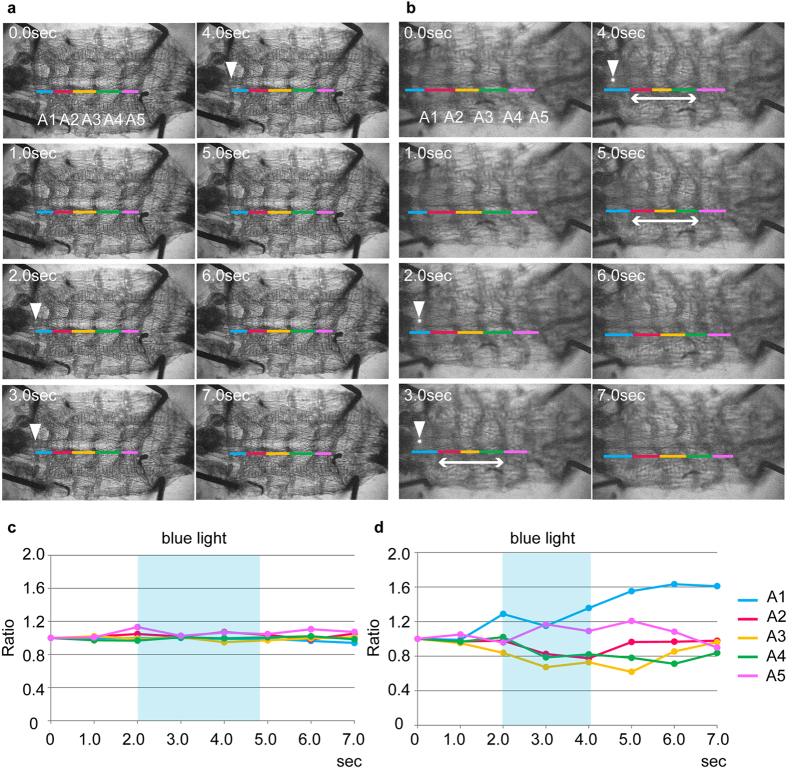
Focal activation of CLIs induces local muscle contraction. (**a**–**d**) Local activation of CLIs induces muscle contraction in muscle segments roughly corresponding to the illuminated neuromeres. (**a**,**b**) Still images of control (**a**) and experimental (**b**) third instar larvae before, during, and after light stimulation. Location of the light application is shown with triangles. A1 to A5 abdominal segments are shown with colored lines. Contracted body segments are shown with double headed arrows (**b**). (**c**,**d**) Ratio of each abdominal segment (A1–A5) before, during, and after the light application is plotted. Muscle length at each time point was divided with the segment length before light illumination. Control (**c**) and experimental (**d**) larvae. A few muscle segments roughly corresponding to the illuminated neuromeres contracted during blue light application (pale blue rectangles). A1–A5: abdominal segments 1 to 5. Colors of the line correspond to the colors shown in (**a**,**b**).

CLIs form putative excitatory synapses with motoneurons in the same segment. Thus, it is likely that CLIs activate motoneurons locally. To test this possibility, we locally activated CLIs in dissected larvae and asked whether the activation induces local muscle contraction (Fig. 7a–d). Light was applied to an abdominal region containing 2–3 neuromeres in either the anterior or middle portion of the abdominal VNC expressing CsChrimson in CLIs. We applied light when the dissected larvae were at quiescence (not undergoing peristalsis) so that we could detect local muscle contractions. Application of the light to the control larvae had no effect (0% [0/7] of the anterior and 0% [0/11] of middle stimulations; Fig. 7a,c and Supplementary Video S6). By contrast, upon applying the focal light to the CsChrimson expressing larvae, we observed contraction of muscles in 2–3 segments corresponding to the illuminated neuromeres (71.4% [5/7, p < 0.05, Fisher’s exact test] of anterior and 95.2% [20/21, p < 0.001, Fisher’s exact test] of middle stimulations; Fig. 7b,d and Supplementary Video S7). The contraction sometimes continued even after the light application was terminated (Fig. 7b, 5.0 sec). These results are consistent with the idea that CLIs are excitatory premotor interneurons that activate motoneurons locally in each segment.

## Discussion

What are the circuit mechanisms that regulate *Drosophila* larval locomotion? To answer this question, it is necessary first to identify the neuronal components of the circuits. Excitatory inputs are critical for the generation of locomotor rhythms in various animals. However, identities and roles of excitatory interneurons that regulate *Drosophila* larval locomotion are unknown. In the present study, we searched for such excitatory interneurons using calcium imaging and identified CLI1s and CLI2s as candidate interneurons that excite motor neurons. Our anatomical and behavioural studies suggest that these neurons directly activate motoneurons locally in each segment during larval locomotion.

The following four lines of evidence suggest that CLIs are excitatory premotor interneurons: (i) CLIs are activated just before the activation of motoneurons in each segment during fictive locomotion, consistent with their providing excitatory drive to motoneurons. (ii) CLIs express ChAT, which synthesizes acetylcholine, a neurotransmitter known to excite motor neurons in this system^25^. (iii) CLIs form GRASP-positive contacts with motoneurons. (iv) Local activation of CLIs results in the contraction of muscles in the corresponding body segments. Although these data are consistent with direct connection between CLIs and motoneurons, it remains possible that CLIs also excite motor neurons indirectly via other interneurons.

CLI1s and CLI2s share many morphological and functional characteristics. i) They are neighboring neurons that send axons along a common path to reach the neuropile. This suggests that they are sibling neurons derived from the same neuroblast^9^,^38^,^39^. Consistent with this notion, they also share the expression of *R47E12-Gal4*. ii) They both project axons along the same fascicle in the anterior commissure and locally innervate motor neurons in the contralateral side of the CNS. iii) They both are cholinergic premotor interneurons and are activated simultaneously during forward locomotion. iv) Activation of these neurons elicits muscle contraction. Taken together, these observations suggest that CLI1s and CLI2s belong to a class of interneurons that fulfill common function(s). There are also distinct features between these two neurons. i) CLI1s innervate the medial neuropile while CLI2s innervate a lateral region, suggesting that they target distinct neurons. ii) CLI1s but not CLI2s project to the next anterior segment. iii) CLI2s are active both during forward and backward locomotion, whereas CLI1s are active only during forward locomotion. Thus, CLI1s only participate in forward locomotion and may activate motor neurons not only in the same segment but also in the next anterior segment, and thus contribute to feed-forward propagation of motor excitation. In contrast, CLI2s may act locally to excite motoneurons only in the same segment and do so both during forward and backward locomotion.

It is currently unknown what motor neurons are the targets of CLI1/2s. Dendrites of motoneurons that innervate different muscle domains form myotopic map along both antero-posterior and medio-lateral axes^40^,^41^,^42^. The axon terminals of CLI1s are located in the medial neuropile, a region occupied by the dendrites of motoneurons innervating ventral muscles. Thus, CLI1s may form synaptic contacts with the ventral motoneurons. Similarly, candidate targets of CLI2s are dorsal motoneurons, since axon terminals of CLI2s are located in a lateral region occupied by these motoneurons. Consistent with this, we observed lifting of the tail, which is likely caused by dorsal muscle contraction when we activated CLI2s but not CLI1s (Supplementary videos S4 and S5). Moreover, CLI1s and CLI2s are activated at a similar timing as aCC in the same segment, a motor neuron that innervates a dorsal muscle and is activated simultaneously with other motor neurons innervating dorsal/ventral internal muscles (Figs 2 and 3). Future studies such as connectomic analyses using serial EM will determine more precisely the downstream circuits of the CLIs.

It is also important to determine in the future the upstream circuits of the CLIs. Since dendritic region of CLI1s and CLI2s partially overlap, these neurons may share common upstream neurons. In particular, because the wave-like activity of CLIs was observed in the isolated CNS that receives no sensory inputs, the activity of CLIs must be regulated by the central circuits that generate a rhythm in an autonomous manner. However, it is also possible that CLIs are activated in response to specific sensory stimulation. Recently, neuronal circuits regulating larval behavior in response to specific sensory stimuli have been identified^23^,^43^. It will be interesting to study the link between these circuits and CLIs.

The wave-like activity of CLIs that occurs concomitant with motor activation strongly suggests that these neurons contribute to sequential activation of motor neurons along the segments during locomotion. Since these neurons are commissural neurons, they may also play a role in left-right coordination, as has been proposed for Dbx1-positive neurons in vertebrates^44^ and recently identified EL neurons in *Drosophila*^22^. However, our loss-of-function analyses thus far failed to reveal roles of CLIs in larval behaviors. We used Shibire^ts^^45^, tetanus toxin light chain^46^, Kir2.1^47^, hid and reaper^48^, and ChAT-RNAi to inhibit the function of CLIs but did not observe any obvious phenotypes. This could be due to insufficient silencing of these neurons by the activity manipulations. It could also be due to the redundancy in the circuit function. It should be noted in this regard that there are likely more CLIs-like neurons present in each segment. The axon terminals of CLI1 and CLI2 only cover part of the motor dendritic region, suggesting other neurons excite motor neurons not targeted by CLIs. Indeed, preliminary results obtained by the ongoing EM reconstruction of the larval CNS suggest that about 10 neurons, in the same neuroblast lineage as CLIs, send their axons locally and contralaterally to the motor region along the common path as CLI1s and CLI2s (Cardona, Truman *et al*., unpublished observation). It is likely that a group of CLIs-like neurons function in a similar manner and together excite the entire motor system. Unfortunately, direct testing of this possibility is not currently feasible due to the unavailability of Gal4 lines specific to this lineage.

Recently, research in our laboratory identified two classes of segmental premotor inhibitory interneurons PMSIs and GVLIs. These neurons are activated slightly later than the motor neurons and appear to inhibit the activity of motoneurons at distinct timings during the motor cycle: PMSIs at an early phase and GVLIs at a final phase of motoneuronal activation^20^,^21^. Here, we identified CLIs that are activated prior to motor neurons and appear to provide an excitatory drive to the motoneurons. These three classes of premotor interneurons likely help shape the pattern of motor activity by providing excitatory and inhibitory inputs to motoneurons at distinct phases of the motor cycle. Since there are only ~400 interneurons per hemisegment in the larval ventral nerve cord, whose connectivity is being reconstructed^43^, we hope that all major classes of premotor interneurons in this system will be identified in the near future. Systematic analyses of CLIs, PMSIs, GVLIs and other premotor neurons will elucidate how the motor patterns generating distinct behaviors are shaped by the combinatorial action of premotor interneurons.

## Materials and Methods

### Fly strains

Fly strains were reared on standard *Drosophila* medium at 25 °C. The following fly strains were used: *w*^*1118*^, *R57C10-Flp2*^32^, *UAS-GCaMP6f*^28^, *UAS-Syt::GFP*^34^, *OK6-LexA*^20^, *UAS-mCD8GFP*^49^, *tsh-Gal80*^30^, *lexAop-CD4::spGFP11*^35^, *UAS-CD4::spGFP1-10*^35^, *UAS-CsChrimson-mVenus*^37^, *R47E12*^27^ (FlyLight, http://flweb.janelia.org/cgi-bin/flew.cgi), *Cha3.3-Gal80*^29^*, pJFRC201-10XUAS-FRT* > *STOP* > *FRT-myr::smGFP-HA, pJFRV240-10XUAS-FRT* > *STOP* > *FRT-myr::smGFP-V5-THS-10XUAS-FRT* > *STOP* >*FRT-myr::smGFP-FLAG*^32^, *RRa-Gal4-F*^31^, split-Gal4 lines: *JRC-SS01256*, *JRC-SS01809*, *JRC-SS01342* (Truman, unpublished results).

### Genetics

For GRASP analysis, the following genetic crosses were used. Experimental group: *OK6-LexA, lexAop-CD4::spGFP11; UAS-CD4::spGFP1-10* were crossed to *OK6-LexA; R47E12-Gal4, Cha3.3-Gal80*. No Gal4 control: *OK6-LexA, lexAop-CD4::spGFP11; UAS-CD4::spGFP1-10* were crossed to *OK6-LexA*. No LexA control: *lexAop-CD4::spGFP11; UAS-CD4::spGFP1-10* were crossed to *R47E12-Gal4, Cha3.3-Gal80*. Parental crosses were reared at 25 °C until dissection of wandering third instar larvae.

### Immunohistochemistry

Immunohistochemistry was performed essentially as described previously^50^. Briefly, we dissected wandering third instar larvae in HEPES buffer (5 mM HEPES, 140 mM NaCl, 2 mM KCl, 6 mM MgCl_2_, 36 mM sucrose) and fixed with 4% formaldehyde in PBS for 30 min. For GRASP analysis, we fixed larvae for 10 min and observed the GFP fluorescent signals. We then incubated the preparation with primary and secondary antibodies for overnight each at 4 °C. The following primary antibodies were used: rabbit anti-GFP (1:1000, Frontier Institute), guinea pig anti-GFP (1:1000, Frontier Institute), rabbit anti-HA (1:1500, Cell Signaling Technology). The following monoclonal antibodies were provided by the Developmental Studies Hybridoma Bank (DSHB): mouse anti-ChAT (1:50), mouse anti-Fas2 (1:10). The secondary antibodies used were anti-mouse Alexa Fluor 555, anti-rabbit Alexa Fluor 488, anti-rabbit Cy3, anti-guinea pig Alexa Fluor 488 (Life Technologies). For fluorescent imaging, we used confocal scanning microscope (Fluoview FV1000, Olympus) with water immersion objective 20X and 60X lenses. Confocal Z-stacks were acquired at 2.5 um interval using 20X, and 0.98 um interval using 60X objective lenses. Frame sizes of the images were 1024 × 768 pixels. The images were processed and analysed using Fluoview (Olympus) and Imaris (Bitplane) software.

### Calcium imaging

Third instar wandering larvae expressing GCaMP6f in neurons of interest were dissected in TES buffer (135 mM NaCl, 5 mM KCl, 4mMMgCl_2_, 2 mM CaCl_2_, 5 mM TES (N-Tris[hydroxymethyl]methyl–2-aminoethanesulfonic acid), 36 mM sucrose)^51^ and immobilized with double-faced tape (Nichiban). Imaging was performed with EMCCD camera (iXon; Andor) mounted on Axioskop2 FS microscope (Zeiss) with a 40X water-immersion objective lens and a spinning-disk confocal unit (CSU21; Yokogawa) at a rate of 10 Hz at 25 °C. Images were analysed using FIJI software^52^. Region of interests (ROIs) were manually drawn around neurites and mean intensity was calculated. Mean intensity of each ROI was normalized and represented as ∆F/F0. F0 was calculated as an average intensity of the first 10 frames with no activity. We applied a look-up table “Royal” to stilled images depicting activities at certain time points to present as pseudocoloured images using FIJI.

### Analysis of calcium imaging data

To calculate the time difference between aCC and CLIs, we first smoothed the fluorescence signal by averaging the intensity over 100 frames (10 seconds). For each time point t, we defined the baseline as the minimum fluorescence signal from t − 10 to t + 10. An event of neural activation was defined as a train of time points in which fluorescence intensities were above (maximum intensity − minimum intensity) ∙ 1/3. To take into account the shapes of the peaks, the time point at which the fluorescence intensity reached half maximum during an event of activation was calculated using Octave^53^ and was used to determine the time difference between the activity of the two neurons. To analyze the phase relationship, activity timings were normalized using the following equation. P_CLI (An)_ = (t_CLI (An)_ − t_aCC (An + 1)_)/(t_aCC (An)_ − t_aCC (An + 1)_). The activities of aCC in the same segment and the next posterior segment were set as 100% and 0% respectively.

### Optogenetic activation

Larvae were reared on standard medium at 25 °C until L2 stage and transferred to an agar plate with yeast paste containing 1 mM all-trans retinal the day before the experiment. Experiments were done at ~25 °C under dim light. Third instar larvae were washed in water to remove yeast paste and acclimated on an agar plate and then used for activation experiment. We recorded moving larvae with CCD camera (XCD-V60, Sony) mounted on stereoscopic microscopy (SZX16 or MVX10; Olympus) at a frame rate of 15 or 30 frames per second. We stimulated behaving larvae with red light (660 nm, LED, Thorlabs) at an intensity of around 30–40 uW/mm^2^. We first recorded for 20 seconds without light and then stimulated with light for 20 seconds followed by 20 seconds without light. We used progeny larvae from *w*^*1118*^ crossed to UAS effector line as a control. For each genotype, 10 larvae were examined. To statistically analyse the data showing percentage, we used one-sided Fisher’s exact test using R software (https://www.r-project.org) and performed Bonferroni correction.

### Local stimulation

Third instar larvae were dissected in TES buffer and kept in a dark place for 5 minutes. To locally activate VNC, we applied, under a confocal microscopy (Fluoview FV1000, Olympus), 488 nm laser light to a region spanning 2–3 neuromeres in the anterior or middle portion of the abdominal VNC in dissected larvae with the body wall. We recorded muscle movements with CCD camera (XCD-V60, Sony) mounted on the confocal microscopy. The length of each muscle segment was measured manually using FIJI software and normalized by dividing the muscle length at each time point by segment length before light illumination.

## Additional Information

**How to cite this article**: Hasegawa, E. *et al*. Identification of excitatory premotor interneurons which regulate local muscle contraction during *Drosophila* larval locomotion. *Sci. Rep.* **6**, 30806; doi: 10.1038/srep30806 (2016).

## Supplementary Material

Supplementary Information

Supplementary Video S1

Supplementary Video S2

Supplementary Video S3

Supplementary Video S4

Supplementary Video S5

Supplementary Video S6

Supplementary Video S7
