# Supplementary material for: Identification of excitatory premotor interneurons which regulate local muscle contraction during Drosophila larval locomotion
Source: Sci Rep. 2016 Jul 29;6:30806. doi: 10.1038/srep30806 (PMC4965782; doi:10.1038/srep30806)
Supplement: Supplementary Information [file srep30806-s1.pdf]

Identification of excitatory premotor interneurons which regulate local muscle contraction during *Drosophila* larval locomotion

Eri Hasegawa, James W. Truman and Akinao Nose

Supplementary Figure S1

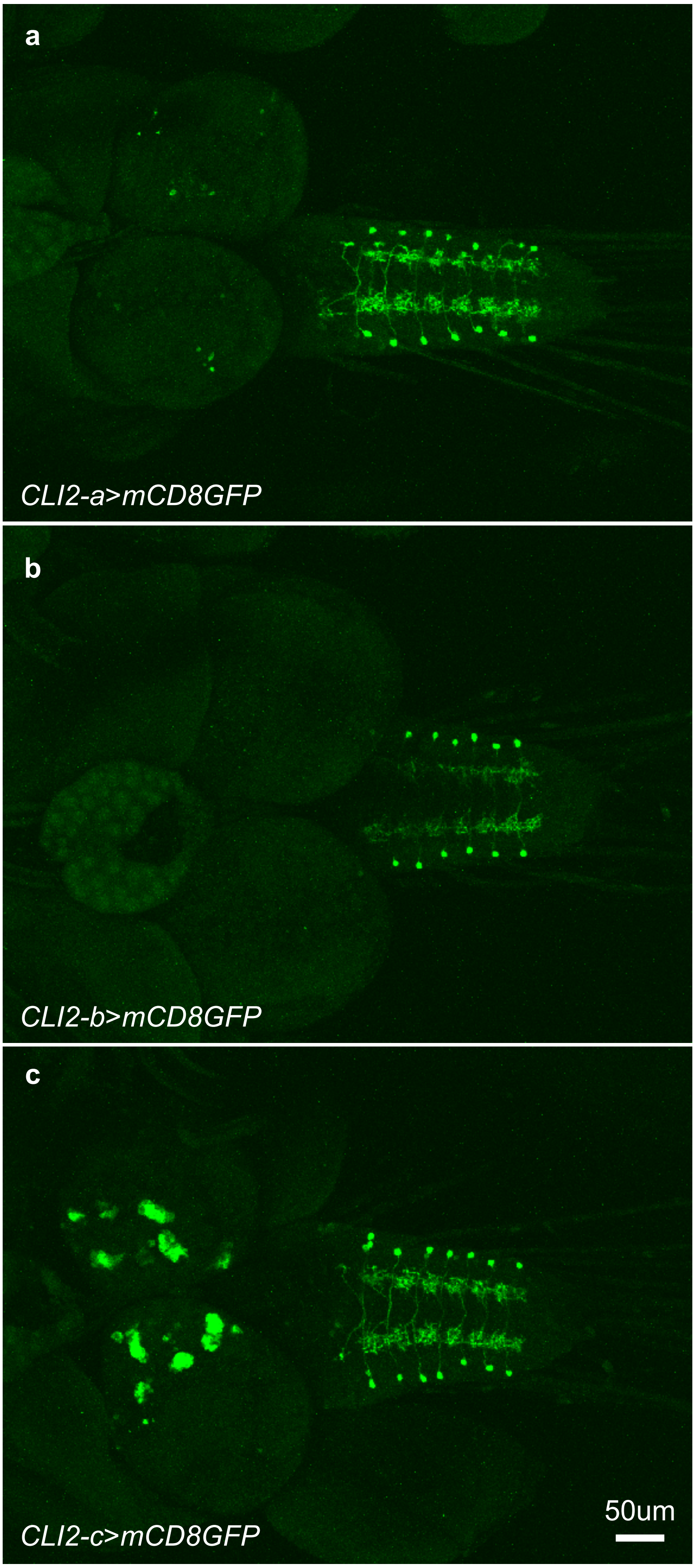

Figure S1. Split-Gal4 lines which are specifically expressed in CLI2s

(a-c) Three split *CLI2-Gal4* lines drive expression specifically in a pair of CLI2s in each abdominal segment (as visualized with *UAS-mCD8GFP*). *CLI2-Gal4-a* (*JRC-SS01256*) (a), *CLI2-Gal4-b* (*JRC-SS01809*) (b), *CLI2-Gal4-c* (*JRC-SS01342*) (c).

Supplementary Figure S2

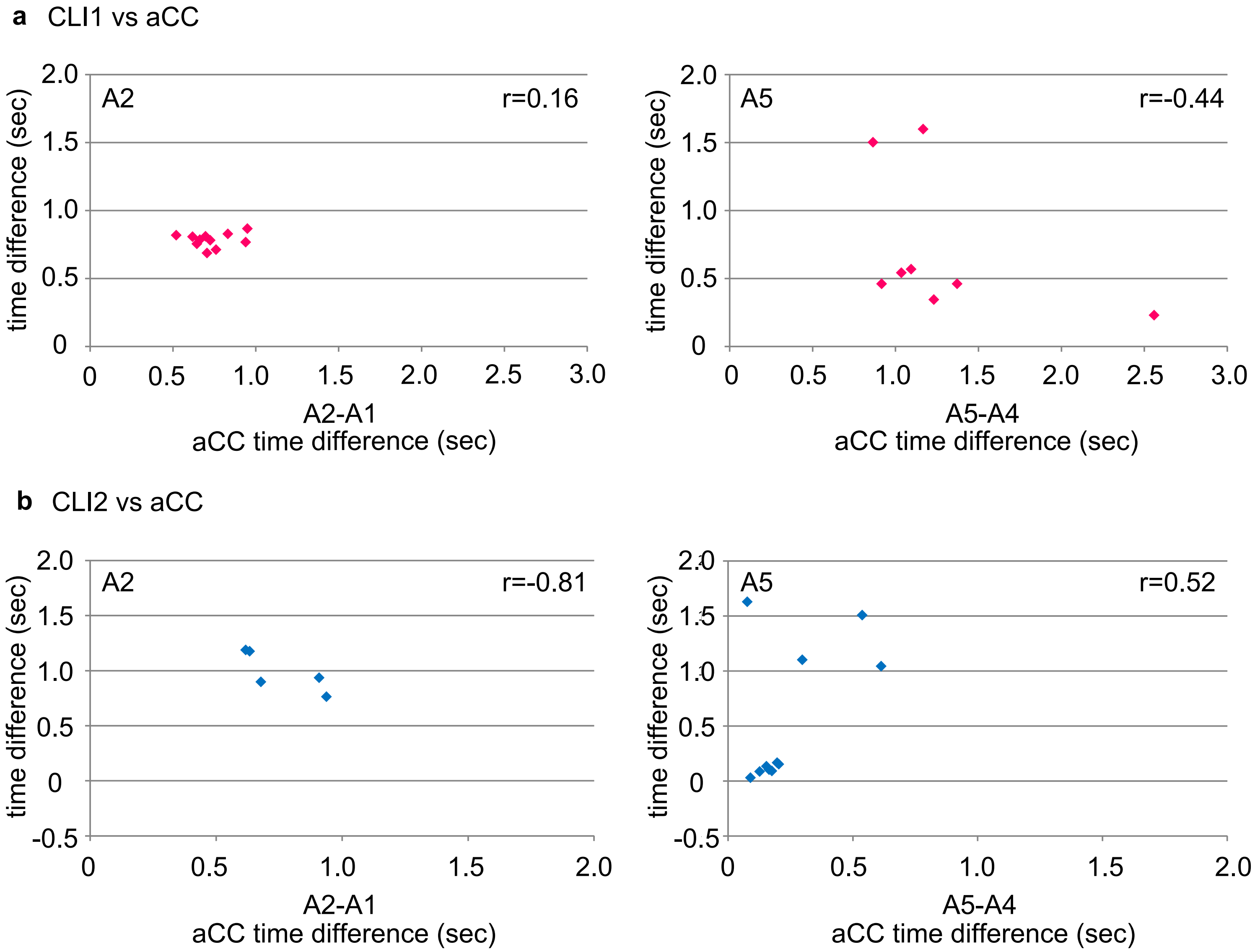

Figure S2. CLI1/2 are activated just prior to aCCs in the same segment

(a, b) Time differences between the activities of CLI1 and aCC (a) and CLI2 and aCC (b) are plotted against the intersegmental time delay. A2 segment (left) and A5 segment (right). (a) 11 waves from 2 larvae (A2), and 8 waves from 3 larvae (A5). (b) 6 waves from 1 larva (A2), and 11 waves from 3 larvae (A5).

Supplementary Figure S3

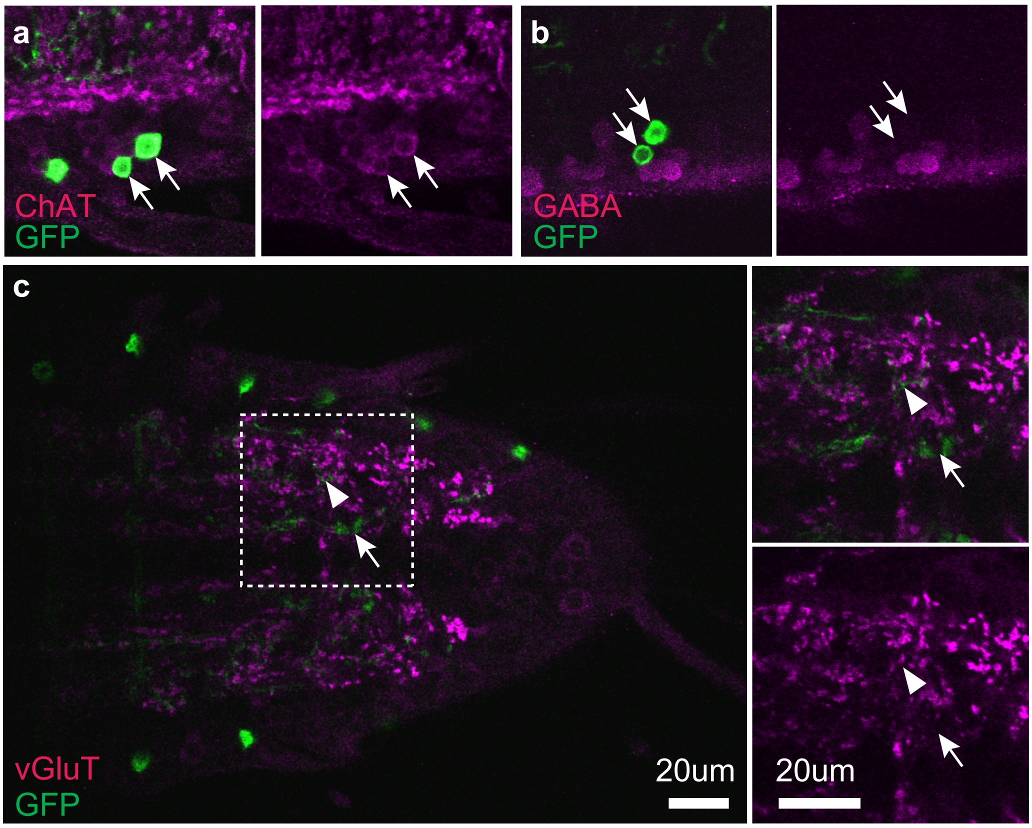

Figure S3. CLI1/2 express ChAT but not GABA or vGluT

(a-c) CLI1/2 were stained with anti-ChAT (a), anti-GABA (b) or anti-vGluT (c) antibodies. The expression of ChAT was observed in cell bodies of CLI1/2 (a) but not that of GABA (b). The cell bodies of CLI1/2 are indicated with arrows. (c) vGluT expression was not oberved in terminals of CLI1/2. The region shown in a square is enlarged in the right panels. Regions corresponding to CLI1s and CLI2s are shown with arrows and triangles respectively.

Supplementary Figure S4

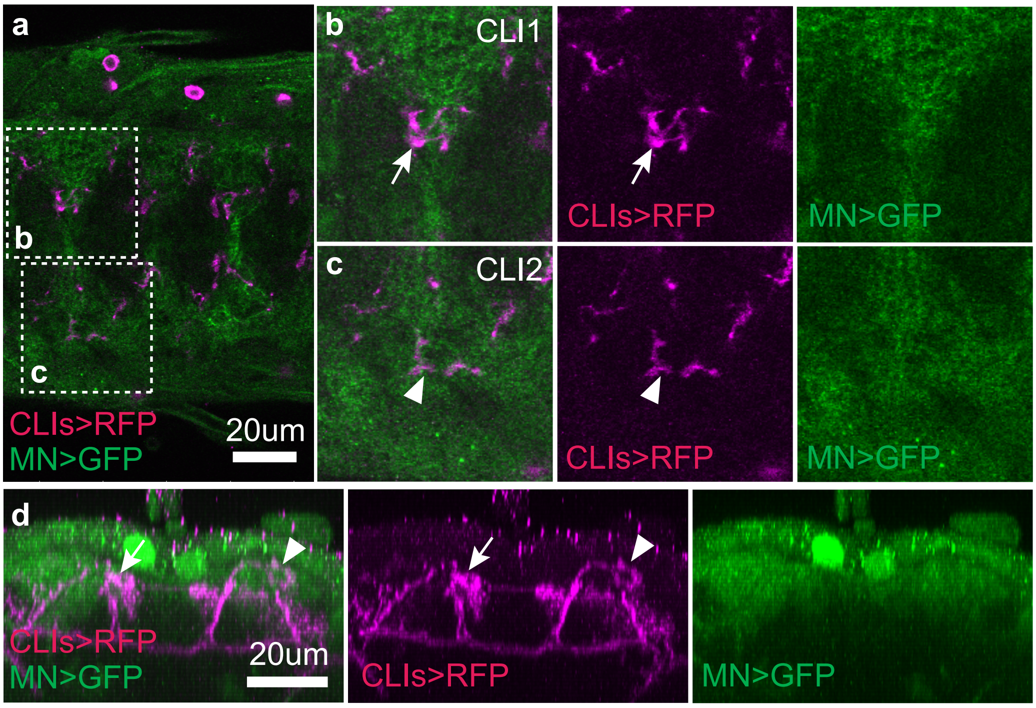

Figure S4. Neurites of CLIs are observed in dorsal regions innervated by dendrites of motoneurons (a-d) CLIs and motoneurons were visualized by expressing mCD8RFP with *CLIs-Gal4* and mCD8GFP with *OK6-LexA*. The regions shown in squares are enlarged in (b) and (c). A cross-sectional view of (a) is shown in (d). Regions corresponding to CLI1s and CLI2s are shown with arrows and triangles respectively.

Supplementary videos

Related to Figure1. CLIs were activated during muscle contraction wave

CLIs>GCaMP6f (Supplementary Video S1)

Related to Figure5. Activation of CLIs using CsChrimson

w X *UAS-CsChrimson* (Supplementary Video S2)

CLIs-Gal4 X *UAS-CsChrimson* (Supplementary Video S3)

CLI1-Gal4 X *UAS-CsChrimson* (SupplemenryVideo S4)

CLI2-Gal4 X *UAS-CsChrimson* (Supplementaary Video S5)

Related to Figure6. Local activation of CLIs using CsChrimson

w X *UAS-CsChrimson* (Supplementary Video S6)

CLIs-Gal4 X *UAS-CsChrimson* (Supplementary Video S7)
